# Outcomes and considerations for retrograde intrarenal surgery (RIRS) in the setting of multiple and large renal stones (>15 mm) in children: Findings from multicentre and real‐world setting

**DOI:** 10.1002/bco2.357

**Published:** 2024-03-31

**Authors:** Patrick Juliebø‐Jones, Vineet Gauhar, Ee Jean Lim, Olivier Traxer, Yesica Quiroz Madarriaga, Daniele Castellani, Khi Yung Fong, Anna Bujons, Deepak Ragoori, Anil Shrestha, Chandra Mohan Vaddi, Tanuj Paul Bhatia, Cagri Akin Sekerci, Yiloren Tanidir, Jeremy Yuen‐Chun Teoh, Bhaskar Kumar Somani

**Affiliations:** ^1^ Department of Clinical Medicine University of Bergen Bergen Norway; ^2^ Department of Urology Haukeland University Hospital Bergen Norway; ^3^ Department of Urology University Hospital Southampton Southampton UK; ^4^ Department of Urology Ng Teng Fong Hospital Singapore Singapore; ^5^ Department of Urology Singapore General Hospital Singapore Singapore; ^6^ Department of Urology Hôpital Tenon Sorbonne University Paris France; ^7^ Urology Department Autonomous University of Barcelona, Fundació Puigvert Barcelona Spain; ^8^ Urology Division, Azienda Ospedaliero‐Universitaria Ospedali Riuniti Di Ancona Università Politecnica Delle Marche Ancona Italy; ^9^ Yong Loo Lin School of Medicine National University of Singapore Singapore Singapore; ^10^ Department of Urology Asian Institute of Nephrology and Urology, Banjara Hills Hyderabad India; ^11^ Department of Urology, National Academy of Medical Sciences Bir Hospital Kathmandu Nepal; ^12^ Department of Urology Preeti Urology and Kidney Hospital Hyderabad India; ^13^ Department of Urology Sarvodaya Hospital Sarvodaya Guwahati India; ^14^ Department of Urology Marmara University School of Medicine Istanbul Turkey; ^15^ Department of Surgery, S.H. Ho Urology Centre The Chinese University of Hong Kong Sha Tin Hong Kong, China

**Keywords:** paediatric, renal stone, retrograde intrarenal surgery, ureteroscopy, urolithiasis

## Abstract

**Purpose:**

The aim of this study is to evaluate the outcomes of retrograde intra renal surgery (RIRS) in the setting of large or multiple stones in children (<18 years).

**Materials and Methods:**

Retrospective analysis was performed of paediatric RIRS cases at nine centres worldwide over a 6‐year period. Patients were divided into two groups: Group 1 had a single stone <15 mm. Group 2 had either multiple stones, maximum stone diameter of >15 mm, or both. Outcomes included stone free rate (SFR) and complications within 30 days.

**Results:**

In total, 344 patients were included with 197 and 147 in Groups 1 and 2, respectively. Ureteric access sheaths were more frequently used in Group 2 (39.5% vs. 56.8%, *p* = 0.021). The operation time was significantly longer in Group 2 (*p* < 0.001). SFR after a single procedure was 84.7% in Group 1 and 63.7% in Group 2. Overall complication rates in Groups 1 and 2 were 7.6% and 33.3%, respectively. The most frequently reported complication in both groups was post‐operative fever (4.4% vs. 14%, *p* = 0.004). The rate of Clavien I/II complications in groups 1 and 2 was 6% and 25.1%, respectively (*p* < 0.05). The rate of Clavien ≥ III complications in groups 1 and 2 was 1.6% and 8.1%, respectively (*p* < 0.05). On multivariate analysis, total operation time, stone size and multiplicity were significant predictors of residual fragments.

**Conclusions:**

RIRS can be performed in paediatric cases with large and multiple stone burdens, but the complication rate is significantly higher when compared to smaller stones.

## 1 INTRODUCTION

The incidence of urolithiasis in the children has increased worldwide, and this has been reflected in the volume of endourology procedures performed in this population group.^1^ For larger stone burden, percutaneous nephrolithotomy (PCNL) has been the traditional intervention of choice.^2^ The principal reason for this is that it offers the highest single‐stage stone free rate (SFR), especially in a large stone volume.^3^ While PCNL is recognized to yield such treatment success, concerns persist surrounding the associated morbidity profile. To this end, retrograde intrarenal surgery (RIRS) is increasingly considered as a feasible alternative.^4^ It can also be a preferred choice in certain complex scenarios.^5^

While there are an increasing number of series reporting outcomes associated with RIRS in children, it is limited in the setting of large and multiple stones. The small body of studies in this particular field is largely limited to single centre and single surgeon series with a relatively small sample size.^6^, ^7^ The aim of this study was to evaluate the outcomes of RIRS in this special clinical scenario of large (>15 mm) or multiple stones in the paediatric age group.

## 2 METHODS

This was a retrospective analysis of paediatric cases of RIRS in nine centres worldwide between January 2015 and December 2020.^6^ Inclusion criteria were paediatric patients (age < 18 years) who underwent RIRS with holmium or thulium fibre laser (TFL) for treatment of renal stones with preoperative non‐contrast computed tomography (NCCT) evaluation. Exclusion criteria were concomitant ureteral stone treatment and bilateral procedures. Patients with missing data were also excluded. Large stones were defined as maximal diameter exceeding 15 mm.

Ethics approval was obtained from the Audit Committee or Institutional Ethics Committee of each participating centre. Data were anonymized before pooled analysis and an Ethics Board approval to analyse the anonymized data was obtained (AINU06/2021).

Patients were divided into two groups: Group 1 consisted of patients with a single stone <15 mm in diameter. Group 2 consisted of patients with either multiple stones, maximum stone diameter of >15 mm, or both. Baseline characteristics and intraoperative and postoperative data were collected from respective institutional databases.

The primary outcome of interest was the incidence of residual fragments and hence stone free rate (SFR) after RIRS. Residual fragments and criteria for stone free status were defined as single fragment >2 mm or multiple fragments of any size assessed at 3 months and evaluated according to local imaging protocols (plain X ray, ultrasound or NCCT). This also served as the definition applied for SFR. Secondary outcome was immediate post‐operative complications (within 30 days).

Continuous variables were presented as means and standard deviations. Categorical data were presented as absolute numbers and percentages. Student's *t*‐test was employed for continuous variables. Chi‐square and Fisher's exact tests were applied for categorical variables. Multivariate regression analysis was used to identify predictors of residual fragments. All analyses were performed in R‐4.3.0. *p*‐value < 0.05 was considered statistically significant.

## 3 RESULTS

In total, 344 patients were included with 197 and 147 in Groups 1 and 2 (Table 1), respectively. The latter group included a higher proportion of recurrent stone formers (8.7% vs. 19.4%, *p* = 0.009), but otherwise, there were no differences for the other baseline characteristics including gender, height and weight. The mean stone sizes were 8.8 mm in Group 1 and 12.8 mm in Group 2. A total of 81.6% of the stones in Group 2 were multiple. There was no difference in terms of pre‐operative features such as rates of positive urine culture, pre‐stenting, stone density and renal anatomy. The renal pelvis was the commonest stone location for Group 1 (52.9%), while it was lower pole for Group 2 (45%).

**TABLE 1:** Baseline characteristics of included patients.

	Group 1 Single, <15 mm (*N* = 197)	Group 2 Multiple or >15 mm (*N* = 147)	*p*
Age, mean (SD)	9.21 (4.65)	9.87 (4.78)	0.203
Males, *n* (%)	120 (60.9)	81 (55.1)	0.331
Height in cm, mean (SD)	125.28 (27.74)	126.86 (29.97)	0.661
Weight in kg, mean (SD)	33.68 (19.49)	35.37 (18.28)	0.441
Presenting symptoms, *n* (%)			
Haematuria only	20 (11.0)	25 (18.0)	0.104
Loin pain only	78 (42.9)	54 (38.8)	0.543
Haematuria and pain	35 (35.7)	19 (29.2)	0.489
Fever only	35 (19.1)	31 (22.5)	0.553
Elevated creatinine, *n* (%)	3 (1.6)	4 (2.9)	0.710
Pre‐op urine culture positive, *n* (%)	37 (19.0)	29 (20.0)	0.922
Recurrent stone former, *n* (%)	16 (8.7)	27 (19.4)	0.009
Pre‐stented, *n* (%)	94 (47.7)	78 (53.1)	0.383
Preoperative tamsulosin, *n* (%)	9 (4.8)	7 (4.9)	>0.99
Preoperative antibiotics, *n* (%)	121 (64.4)	100 (69.4)	0.392
Normal kidney anatomy, *n* (%)	169 (85.8)	120 (81.6)	0.373
Largest stone diameter (mm), mean (SD)	8.81 (2.51)	12.8 (5.68)	<0.001
Stone type, *n* (%)			<0.001
Single	197 (100)	27 (18.4)	
Multiple	0	120 (81.6)	
HU, mean (SD)	895.12 (323.72)	847.11 (374.70)	0.369
Stone location, *n* (%)			
Upper pole	24 (12.2)	43 (29.7)	<0.001
Middle pole	34 (17.3)	62 (42.8)	<0.001
Lower pole	53 (27.0)	84 (57.9)	<0.001
Renal pelvis	27 (52.9)	18 (45.0)	0.589

Abbreviation: HU, Hounsfield units.

Regarding intra‐operative characteristics, ureteric access sheath (UAS) was more frequently used in Group 2 (39.5% vs. 56.8%, *p* = 0.021) (Table 2). There were no differences in terms of laser type, fragmentation strategy or rates of post‐operative stenting between the groups. Total operation time was longer in Group 2 (28.04 min vs. 32.83 min, *p* < 0.001).

**TABLE 2:** Intraoperative characteristics.

	Group 1 Single, <15 mm (*N* = 197)	Group 2 Multiple or >15 mm (*N* = 147)	*p*
Ureteric dilation to accommodate UAS, *n* (%)	20 (10.2)	11 (7.5)	0.506
UAS > 8 Fr, *n* (%)	60 (39.5)	42 (56.8)	0.021
Distal scope size (Fr), *n* (%)			0.007
<7 Fr	24 (23.5)	15 (14.4)	
7.5 Fr	56 (54.9)	46 (44.2)	
8 Fr and above	22 (21.6)	43 (41.3)	
Reusable fURS, *n* (%)	190 (96.4)	143 (97.3)	0.901
Stone clearing technique, *n* (%)			
Dusting, *n* (%)	71 (36.0)	72 (49.0)	0.022
Popcorning, *n* (%)	3 (1.5)	3 (2.0)	>0.99
Dusting + popcorning, *n* (%)	123 (62.4)	77 (52.4)	0.078
Thulium fibre laser, *n* (%)	7 (4.1)	5 (3.9)	>0.99
Basket extraction, *n* (%)	14 (20.0)	12 (31.6)	0.268
Postoperative stenting, *n* (%)	33 (41.8)	16 (47.1)	0.754
Fragmentation time (min), mean (SD)	45.93 (128.15)	50.39 (92.19)	0.841
Total operation time (min), mean (SD)	58.18 (28.04)	84.64 (32.83)	<0.001

In terms of treatment success, the rate of residual fragments was lower in Group 1 (15.3% vs. 55.3%, *p* < 0.001). The rate of re‐intervention was higher in in Group 2 (19.8% vs. 37.5%, *p* = 0.003). Repeat RIRS was the commonest second procedure to be selected. SFR after a single procedure was 84.7% in Group 1 and 63.7% in Group 2.

The overall complications rates in Group 1 and 2 were 7.6% (*n* = 15) and 33.3% (*n* = 49) (*p* < 0.05), respectively. The rate of Clavien I/II complications in groups 1 and 2 was 6% and 25.1%, respectively (*p* < 0.05). The rate of Clavien ≥ III complications in groups 1 and 2 was 1.6% and 8.1%, respectively (*p* < 0.05) (Table 3). The most frequently reported complication in both groups was post‐operative fever requiring medication (4.4% vs. 14%, *p* = 0.004). This was followed by postoperative haematuria persisting for 24 h but not needing transfusion (1.6% vs. 9.8%, *p* = 0.003). Clavien III complication included sepsis needing intensive care unit (ICU) admission in two patients each and 10 patients in group 2 who needed ureteric stent due to pelvicalyceal or ureteric injury detected on check retrograde pyelogram at the end of the procedure or direct visualization.

**TABLE 3:** Postoperative complications, expressed in *n* (%).

	Group 1 Single, <15 mm (*N* = 197)	Group 2 Multiple or >15 mm (*N* = 147)	*p*
Bleeding not needing transfusion	1 (0.7)	3 (2.4)	0.508
Pelvicalyceal system injury needing stenting	0 (0.0)	5 (3.5)	0.037
Ureteric injury due to UAS, needing stenting	1 (0.5)	5 (3.5)	0.123
Postoperative fever needing medication	8 (4.4)	20 (14.0)	0.004
Postoperative haematuria persisting for 24 h but not needing transfusion	3 (1.6)	14 (9.8)	0.003
Postoperative sepsis, needing ICU	2 (1.1)	2 (1.4)	>0.99
Residual fragment (single > 2 mm or multiple)	26 (15.3)	73 (55.3)	<0.001
Reintervention for residual fragment	25 (19.8)	45 (37.5)	0.003
ESWL	0 (0.0)	2 (1.4)	0.373
RIRS	24 (13.1)	39 (27.3)	0.002
PCNL	1 (0.5)	5 (3.5)	0.119
ECIRS	0 (0.0)	3 (2.1)	0.166

On univariate analysis, lower power setting, total operation time, stone size and multiplicity were significant predictors of residual fragments (Table S1). On multivariate analysis, significance was only found for total operation time, stone size and multiplicity (Table S2).

## 4 DISCUSSION

RIRS can be performed in children with larger stone burden, but the complication rate and need for repeat intervention is significantly higher. Of note, the majority (75.5%) of complications in the large stone group were minor in nature. The increasing trend towards application of RIRS in paediatric cases has been largely influenced by technological advances found with newer generations of scopes, as well as the energy sources employed for stone lithotripsy.^8^ This included the development of smaller dimension ureteroscopes, which both allow for reduced likelihood for pre‐stenting and allowing for a smaller sized UAS. The latter has attracted caution previously given risk of complications such as ureteric injury and stricture.^9^ The use of UAS was higher in Group 2, and this could have contributed to the higher complication burden in this group and in particular, the higher rate of pelvicalyceal injury. RIRS can also be a more feasible option in geographic settings where paediatric endourology may not be a defined subspeciality and such cases are performed by adult urologists.^10^ In such setting, the learning curve for RIRS in children is shorter compared to PCNL.^11^ Regardless of choice of RIRS or PCNL, even once the learning curve has been reached, a higher volume of paediatric cases has been found to deliver improved outcomes.^12^ This is an additional challenge when the incidence of paediatric urolithiasis is relatively low.

As well as the steeper learning curve, standard (maxi) PCNL is associated with a higher rate of serious complications compared to RIRS, the most noteworthy being bleeding.^13^ However, miniaturization of PCNL equipment offers an additional intervention choice that is now available and can be associated with an improved safety profile compared to standard PCNL. A recent study comparing super mini PCNL and RIRS in children with stone burdens of 1–2 cm found the former to yield superior SFRs as well as a lower complication rate.^14^ To this end, miniaturized PCNL appears to represent an increasingly favourable option for this patient group especially. Depending on resources, expertise as well as individual patient factors such as anatomy, miniaturized PCNL may not be a treatment option for a select case and therefore RIRS may be employed. Surgeons should therefore be aware of the potential for higher rate of complications and observe closely in the post‐operative period accordingly.

## 5 STRENGTHS AND LIMITATIONS

This study offers one of the largest series on paediatric stones including for large and multiple stones, where studies have generally been lacking. It is strengthened by its multicentre status with data collected from nine subspecialty centres across the world. In contrast, the majority of previous multicentre centres have been limited to data from two or three centres only.^15^, ^16^ However, it is retrospective in nature, and no comparison has been made to alternative interventions such as PCNL. Thulium fibre laser (TFL) is a newer laser platform available in clinical practice, and several studies have concluded it can deliver higher SFRs.^17^ This study only had a limited number of patients using TFL. However, as in our series, Holmium laser remains the commonest laser in use worldwide, and therefore, the results may be more generalizable to a real‐world setting as a result. While all patients had NCCT to determine pre‐operative stone burden, different modalities were used to determine stone clearance according to local protocols and this is a limitation.

With paediatric RIRS now getting more acceptable, it is perhaps time for guidelines to update the management algorithms.^18^ Clinicians must also make use of treatment nomograms to help with their decision making.^19^ This study lacks the ability to comment on laser settings. The importance of this is increasingly being recognized alongside the need for operator awareness regarding of intra‐renal pressure and temperature.^13^, ^18^, ^19^ This applies to RIRS in children as well. Future studies evaluating the use of suction devices during RIRS will be of interest in improving SFR and minimizing need for secondary interventions.^20^

## 6 CONCLUSION

RIRS can be performed in paediatric cases with large and multiple stone burdens, but the complication rate is significantly higher when compared to smaller stones. The majority of these complications are, however, minor in severity. Close post‐operative observation is therefore of even higher importance in this patient group. Parents must also be counselled about the need for a staged procedure for complete stone clearance in such cases.

## AUTHOR CONTRIBUTIONS

**Patrick Juliebø‐Jones:** Data collection, writing—draft, writing, revision, planning. **Vineet Gauhar:** Project development, data collection, supervision, methodology, writing—revision. **Ee Jean Lim:** Project development, data collection, analysis, writing—revision. **Olivier Traxer:** Project development, data collection, analysis, writing—revision. **Yesica Quiroz Madarriaga:** Project development, data collection, analysis, writing—revision. **Daniele Castellani:** Data analysis and collection, writing—revision, data management. **Khi Yung Fong:** Data collection, analysis, writing—revision. **Anna Bujons:** Data collection, data collection, analysis, writing—revision. **Deepak Ragoori:** Data collection, analysis, writing—revision. **Anil Shrestha:** Data collection, analysis, writing—revision. **Chandra Mohan Vaddi:** Data collection, analysis, writing—revision. **Tanuj Paul Bhatia:** Data collection, analysis, writing—revision. **Cagri Akin Sekerci:** Data collection, analysis, writing—revision. **Yiloren Tanidir:** Data collection, analysis, writing—revision. **Jeremy Yuen‐Chun Teoh:** Data collection, analysis, writing—revision. **Bhaskar Kumar Somani:** Supervision, planning, writing—revision, data collection.

## CONFLICT OF INTEREST STATEMENT

The authors have none to declare.

## Supporting information


**Table S1.** Univariate analysis of predictors of residual fragments

**Table S2.** Multivariate analysis of predictors of residual fragments

## Data Availability

The data sets generated during and/or analysed during the current study are available from the corresponding author on reasonable request.
