# Supplementary material for: Outcomes and considerations for retrograde intrarenal surgery (RIRS) in the setting of multiple and large renal stones (>15 mm) in children: Findings from multicentre and real‐world setting
Source: BJUI Compass. 2024 Mar 31;5(6):558–63. doi: 10.1002/bco2.357 (PMC11168767; doi:10.1002/bco2.357)
Supplement: Supplementary file 2 — Table S2. Multivariate analysis of predictors of residual fragments [file BCO2-5-558-s001.docx]

Supplementary Table 2: Multivariate analysis of predictors of residual fragments

| *Predictors* | *Odds Ratio* | *95%CI* | *p* |
| --- | --- | --- | --- |
| Age | 1.012 | 0.963-1.063 | 0.637 |
| Total operation time | 0.982 | 0.974-0.99 | **<0.001** |
| Largest stone diameter | 0.895 | 0.845-0.944 | **<0.001** |
| Stone HU | 1.000 | 0.999-1.001 | 0.936 |
| Low-power (vs High-power) | 0.328 | 0.179-0.574 | **<0.001** |
| Male gender | 1.213 | 0.757-1.941 | 0.422 |
| Prestented | 1.242 | 0.779-1.988 | 0.363 |
| Prestented >14 days | 1.294 | 0.735-2.322 | 0.378 |
| Multiple stone (compared to single) | 0.241 | 0.146-0.394 | **<0.001** |
| UAS >8Fr | 0.631 | 0.334-1.193 | 0.154 |
| Reusable fURS | 0.544 | 0.08-2.299 | 0.453 |
| Postoperative stenting | 0.540 | 0.245-1.178 | 0.123 |

HU= Hounsfield units

UAS = Ureteral access sheath

Fr = French

fURS = flexible ureteroscope
